# Supplementary material for: Content-rich biological network constructed by mining PubMed abstracts
Source: BMC Bioinformatics. 2004 Oct 8;5:147. doi: 10.1186/1471-2105-5-147 (PMC528731; doi:10.1186/1471-2105-5-147)
Supplement: Additional File 2 — The original results of the above study (non-essential files are deleted to keep the file size under the limit set by BMC bioinformatics). [file 1471-2105-5-147-S2.bz2 › chilibotAdditionalFile2/dip20/696ID11722740E18021/html/TRAK_TRAB.html]

 


 **TRAK** and **TRAB** 
  
Found 13 abstracts in PubMed, retrieved 13.  
 

 What does Google say? 
 PDF only 
| .edu only 

---

**Interactive relationship** (e.g. stimulation, inhibition, etc)

**Neutral relationship**- A  **traB**  bait exclusively identified  **traK**  preys.  Ref: 11722740 Mol Microbiol, 2001
- Thyrotrophin receptor antibody  **TRAb**  levels were measured using LUMItest  **TRAK**  BRAHMS Diagnostica, Berlin, Germany.  Ref: 11589683 Clin Endocrinol Oxf , 2001
- A  **traK**  bait in turn identified traV and  **traB** , which is predicted to encode an inner membrane protein.  Ref: 11722740 Mol Microbiol, 2001
- **TRAb**  was tested by an isotopic receptor assay  **TRAK**  Henning in 80 newly diagnosed, untreated Graves disease patients group I, 63 with other thyroid diseases group II and 60 controls group III.  Ref: 9005701 Acta Med Port< MedlineTA>Acta Med Port, 2001
- In contrast,  **TraB**  fractionated with the inner membrane in traV or  **traK**  mutant cells, and  **TraK**  appeared in the osmotic shock fluid from the traV mutant.  Ref: 11722740 Mol Microbiol, 2001
- In 122 Graves patients  **TRAb**  were measured at the time of diagnosis and in all patients when discontinuing ATD by a competitive radioreceptor assay using recombinant human TSH receptors  **TRAK**  human assay.  Ref: 11834425 Eur J Endocrinol, 2002
- Further, in  **traK**  mutant cells, TraV failed to accumulate to a detectable level, and the  **TraB**  level was significantly reduced, suggesting that TraV and  **TraB**  must interact with  **TraK**  for either protein to accumulate to its normal level.  Ref: 11722740 Mol Microbiol, 2001

**Non-interactive relationship** (e.g. studied together, co-existance, homology, etc.)

- These results are consistent with a TraV  **TraK**   **TraB**  complex anchored to the outer membrane via the TraV lipoprotein.  Ref: 11722740 Mol Microbiol, 2001
- Despite the higher diagnostic sensitivity of the  **TRAK**  human method, we could not find any improvement of predictive values for relapse of hyperthyroidism in the measurement of  **TRAb**  at the end of ATD.  Ref: 11834425 Eur J Endocrinol, 2002
- First, two hybrid analyses indicated that TraV and  **TraB**  bind to different  **TraK**  segments, as required if  **TraK**  bridges a ternary complex.  Ref: 11722740 Mol Microbiol, 2001
- Evidence that F plasmid proteins TraV,  **TraK**  and  **TraB**  assemble into an envelope spanning structure in Escherichia coli.  Ref: 11722740 Mol Microbiol, 2001
- Mutant analysis demonstrated that this synthesis is independent of host expression of traA, traL, traE,  **traK** ,  **traB** , traV, traW, traC, traU, traF, or traH, but dependent on expression of the traF traH region.  Ref: 6219273 Mol Gen Genet, 1982
- Several additional observations support the hypothesis that TraV,  **TraK**  and  **TraB**  form a complex in Escherichia coli that spans the cell envelope from the outer membrane TraV through the periplasm  **TraK**  to the inner membrane  **TraB** .  Ref: 11722740 Mol Microbiol, 2001
- METHODS In this study we evaluated the diagnostic performance of a newly developed 2nd generation  **TRAb**  assay  **TRAK**  human DYNOtest, BRAHMS Diagnostica GmbH, Berlin, Germany in 74 untreated patients affected by Graves disease, 53 untreated patients affected by Hashimoto s thyroiditis and 88 patients affected by euthyroid nodular goiter.  Ref: 11456370 Q J Nucl Med, 2001
- The aim of this study was to compare two two step assays, a new coated plate CP ELISA assay  **TRAb**  ELISA using purified porcine TSH receptors pTSH R and a coated tube assay CT using recombinant human TSH receptors hTSH R DYNO R test  **TRAK**  human.  Ref: 12733717 Endocr J, 2003
- A polypeptide which migrate in the F pilin position was still present in membranes prepared from Flac strains carrying mutations in traL, traE,  **traK** ,  **traB** , traV, traW, traC, traU, traF, traH or traG despite the inability of these mutants to elaborate F pili filaments.  Ref: 6120442 Mol Gen Genet, 1981
- We also compared the new  **TRAb**  assay with the 1st generation test  **TRAK**  Assay, BRAHMS Diagnostica GmbH, Berlin, Germany and anti thyroperoxidase assay AbTPO DYNOtest, BRAHMS Diagnostica GmbH, Berlin.  Ref: 11456370 Q J Nucl Med, 2001
